# The Antistaphylococcal Activity of Amoxicillin/Clavulanic Acid, Gentamicin, and 1,8-Cineole Alone or in Combination and Their Efficacy through a Rabbit Model of Methicillin-Resistant *Staphylococcus aureus* Osteomyelitis

**DOI:** 10.1155/2020/4271017

**Published:** 2020-04-28

**Authors:** Soukayna Hriouech, Ahmed A. Akhmouch, Aouatef Mzabi, Hanane Chefchaou, Mariam Tanghort, Bouchra Oumokhtar, Najat Chami, Adnane Remmal

**Affiliations:** ^1^Laboratory of Biotechnology, Faculty of Sciences Dhar El Mehraz, University Sidi Mohamed Ben Abdallah, BP 1796 Atlas, 30 050 Fez, Morocco; ^2^Laboratory of Microbiology and Molecular Biology, Faculty of Medicine and Pharmacy, University Sidi Mohammed Ben Abdallah, BP 1893 Route Sidi Harazem, 30 070 Fez, Morocco

## Abstract

The aim of this research paper is to test the antistaphylococcal effect of 1,8-cineole, amoxicillin/clavulanic acid (AMC), and gentamicin, either separately or in combination against three *Staphylococcus aureus* strains isolated from patients suffering from osteomyelitis. This activity was tested *in vitro* by using the microdilution method and the checkerboard assay. The efficacy of these three antibacterial agents was then tested *in vivo* by using an experimental model of methicillin-resistant *S. aureus* osteomyelitis in rabbits. This efficacy was assessed after four days of treatment by counting the number of bacteria in the bone marrow. The obtained results *in vitro* showed that the combination of the AMC with gentamicin did not induce a synergistic effect, whereas the combination of the two antibiotics with 1,8-cineole did. This effect is stronger when AMC is combined with 1,8-cineole as a total synergistic effect was obtained on the three strains used (FIC ≤ 0.5). *In vivo*, a significant reduction was noted in the number of colonies in the bone marrow when rabbits were treated with AMC associated with either 1,8-cineole or gentamicin compared to rabbits treated with AMC, gentamicin, or 1,8-cineole alone. These results demonstrated that 1,8-cineole showed a synergistic effect in combination with both AMC and gentamicin, which offer possibilities for reducing antibiotic usage. Also, the AMC associated with 1,8-cineole could be used to treat MRSA osteomyelitis.

## 1. Introduction

Osteomyelitis is a bacterial infection characterized by an acute or chronic inflammatory response that leads to bone loss. Furthermore, the spread of this infection to surrounding tissues is responsible for significant morbidity and healthcare costs each year [1]. In high-income countries, acute osteomyelitis occurs in about 1of 800,000 children per year [2] but it is considerably more common in low- and middle-income countries [3]. In Morocco, osteomyelitis is more common among children [4].

The most common causative organism of osteomyelitis is *S. aureus* [5, 6]. The antibiotic of choice in the treatment of bone infections is vancomycin. However, the resistance of staphylococci to vancomycin has been reported [7, 8]. New alternatives are becoming essential to overcome the increasing resistance of *S. aureus* strains and to improve the antimicrobial treatment of bone infections. Our laboratory, which has extensively worked on essential oils (EOs) and their major compounds, demonstrated the antimicrobial activity of these components *in vitro* and *in vivo* [9–12]. The advantage of EOs over other antimicrobial agents is that they offer high antibacterial potency without inducing the production of resistance mechanisms [13–15]. Among the various constituents of EOs, 1,8-cineole has been shown to have pharmacological effects. 1,8-cineole has been used as a percutaneous penetration enhancer, an antibacterial expectorant, and an anti-inflammatory agent [16]. 1,8-cineole was reported to induce apoptosis in leukemia cell lines [17]. Additionally, the importance of combining EOs with antibiotics to fight resistant bacteria is increasingly recognized [18]. In fact, this combination has shown a synergistic effect against antibiotic-resistant bacteria [19, 20]. The aim of the present research is to evaluate the *in vitro* antistaphylococcal effect of a major compound of EOs 1,8-cineole and two antibiotics AMC and gentamicin either separately or in combination and to elucidate the *in vivo* efficacy in a rabbit model of methicillin-resistant *S. aureus* (MRSA) osteomyelitis.

## 2. Materials and Methods

### 2.1. Antimicrobial Agents

- Amoxicillin/clavulanic acid (AMC): Augmentin® 1 g/200 mg, in powder form, for injectable solution purchased from GlaxoSmithKline (Morocco) was used. It was dissolved in 10 mL (w/v) of sterile distilled water and stirred until totally dispersed. The final concentration of AMC obtained was 100 mg/mL.
- Gentamycin: Gentosyl® solution for injection at a concentration of 10 mg/mL, purchased from Laprophan (Morocco), was used in this study.
- 1,8-cineole, in liquid form, provided by Sigma-Aldrich (France), was dispersed in a viscous solution of 0.2% (v/v) agar according to the method described by Remmal et al. [21]. The stock solution prepared according to this procedure had a concentration of 100 mg/mL.

### 2.2. Culture Media

Mueller–Hinton agar (MHA, Biokar®), Mueller–Hinton broth (MHB, Biokar®), tryptic soy agar (TSA, Oxoid®), tryptic soy broth (TSB, Oxoid®), and Chapman agar (Biokar®) were prepared and sterilized according to the manufacturers' instructions.

### 2.3. Bacterial Strains and Inoculums Standardization

In this study, the antibacterial activity of each agent and their combination was tested against three bacterial strains: a strain of MRSA and two strains of methicillin-susceptible *S. aureus* (MSSA) (Table 1). The methicillin resistance was determined by the cefoxitin disk diffusion test using 30 mg cefoxitin disks on Mueller–Hinton agar, as recommended by CLSI guidelines [22], and confirmed by the detection of mecA gene by PCR assay [23].

They were isolated from the bone marrow of patients suffering from osteomyelitis and were obtained from the Laboratory of Microbiology and Molecular Biology, Faculty of Medicine and Pharmacy of Fez (Morocco).

Stock cultures were kept on a Muller–Hinton agar under refrigeration (4°C). The inoculum suspension was obtained by taking colonies from 24 h cultures on tryptic soy agar. These colonies were suspended in sterile saline (0.9% NaCl) and shacked for 15 seconds. The density was adjusted to the turbidity of a 0.5 McFarland Standard (equivalent to 1.5 × 10^8^ CFU/mL) [24].

### 2.4. Minimal Inhibitory Concentration (MIC)

The MICs of AMC, gentamicin, and 1,8-cineole were determined by microdilution assays in 96-well plates according to the standards of the CLSI [25]. Ten concentrations of 1,8-cineole and the two antibiotics AMC and gentamycin were prepared in sterile hemolysis tubes by serial dilutions. The concentrations of AMC obtained in the well were between 32 *μ*g/mL and 0.062 *μ*g/mL, between 4 *μ*g/mL and 0.0078 *μ*g/mL for gentamicin, and between 64 mg/mL and 0.125 mg/mL for 1,8-cineole to determine the MIC values. Bacterial suspensions were prepared as previously described. These suspensions were diluted in MH broth and plated in 96-well plates at a density of 5 × 10^5^ CFU/well. After the plates were incubated at 37°C for 18 hours, 40 *μ*L of 0.5% triphenyl-tetrazolium chloride was added to each well. After two hours of incubation, the MIC corresponds to the lowest concentration that does not produce a red color [24].

### 2.5. Checkerboard Assay

The evaluation of the interaction between AMC, gentamicin, and 1,8-cineole was performed according to the method of Mulyaningsih et al. [26]. Briefly, eight concentrations of antibiotics and eight concentrations of 1,8-cineole were prepared in sterile hemolysis tubes by successive dilutions 1/2. For antibiotics, the concentrations were introduced vertically into eight wells in a decreasing manner ranging from MIC × 2 to MIC/64, while the concentrations of 1,8-cineole were introduced horizontally into eight wells in a decreasing manner ranging from MIC × 2 to MIC/64. The combination of AMC and gentamicin was performed in the same way. Each association was performed in duplicate.

The analysis of the combination was obtained by calculating the fractional inhibitory concentration (FIC) index (FICI) using the following formula [27]: FICI = FIC (A) + FIC (B), where FIC (A) = [(MIC (A) in combination/MIC (A) alone)] and FIC (B) = [(MIC (B) in combination/MIC (B) alone)].

The index values of the fractional inhibitory concentrations were interpreted as follows: FICI ≤ 0.5 means synergy; 0.5 < FICI ≤ 0.75 means partial synergy; 0.76 ≤ FICI ≤ 1 means additive effect; 1 < FICI ≤ 4 means no interaction (not differential); FICI > 4 means antagonism.

### 2.6. Animals

Forty-two female New Zealand white rabbits (5–6 weeks old), weighing between 1.2 and 1.8 kg, were used in this study. They were divided into seven groups of six rabbits each. The rabbits were given feed and water *ad libitum* and were treated in accordance with the National Health and Research Council Ethics Committee guidelines. Adequate ventilation was provided, and the environmental temperature was constantly maintained at 21°C ± 3°C. The photoperiod was adjusted daily to 12 h of light and 12 h of darkness. For the purpose of acclimatization, the animals of the experiment were kept for a week. Advice with regard to the surgical procedures was sought from a professional vet and from a surgeon.

### 2.7. Groups of Animals

The animals were randomly divided into seven experimental groups of six rabbits each:

  Group 1 (*n* = 6), positive control group: infected, untreated animals.
  Group 2 (*n* = 6): animals infected and treated with AMC at a dose of 30 mg/kg.
  Group 3 (*n* = 6): animals infected and treated with gentamicin at a dose of 3 mg/kg.
  Group 4 (*n* = 6): animals infected and treated with 1,8-cineole at a dose of 12 mg/kg.
  Group 5 (*n* = 6): animals infected and treated with AMC at a dose of 15 mg/kg associated with gentamycin at a dose of 1.5 mg/kg.
  Group 6 (*n* = 6): animals infected and treated with AMC at a dose of 15 mg/kg combined with 1,8-cineole at a dose of 6 mg/kg.
  Group 7 (*n* = 6), negative control group: neither infected nor treated animals.

The doses administered were calculated according to the weight by imitating the recommended human dose for each drug; 12 mg/Kg given twice daily for 1,8-cineole [28], 31.83 mg/kg/twice daily AMC [29], and 6 mg/kg once daily [30].

### 2.8. Bacterial Strain and Preparation of the Inoculum

Among the three strains studied, *in vitro*, the MRSA strain was chosen for the *in vivo* study. From an overnight culture of MRSA in a 9 mL tryptic soy broth, aliquots of 100 *μ*L were transferred to sterile tubes containing 3 mL of TSB. These tubes were incubated for 3 h at 37°C to obtain log-phase growth [31]. After incubation, the tubes were centrifuged for 10 min at 1000 g, the supernatant was decanted, and the remaining pellet was washed twice with phosphate-buffered saline (PBS). Under spectrophotometric control (McFarland score), the bacterial sediment was added to the PBS. A suspension containing 10^9^ CFU/mL was obtained.

### 2.9. Experimental Design

A fentanyl patch (Durogesic®) was used for the management of pain during the study. Due to the delay in action (about 12 h), the patch was placed the night before the beginning of the experiment (induction) and changed every 72 h. On the first day of the study, which was considered to be day zero (day 0); the rabbits were anesthetized by injection of a mixture of xylazine at 1 mg/kg and ketamine at 20 mg/kg into the marginal vein of the ear, then the right knee of the animal was shaved, and the skin was disinfected with povidone-iodine (Betadine®). We used a percutaneously transarticular route to perform a femoral trepanation using a Jamshidi bone marrow biopsy needle (8 Ga). The Jamshidi needle was inserted between the two femoral condyles and through the epiphysis, physis, and metaphysis to reach the medullary canal. Then, a 1 mL suspension containing 10^9^ CFU/mL of MRSA was injected into the tibia. The procedures used in this experimental model are described by Gaudin et al. [31] and Amador et al. [32]. The infection was allowed to develop for three days.

On the third day, in order to quantify the infection, the rabbits were anesthetized as before, and bone marrow samples were taken using 8 Ga syringes, weighed, and mixed with 200 *μ*L of physiological serum, and the resulting solution was seeded in pure and diluted forms at 10^−2^, 10^−4^, and 10^−6^ on Chapman gel. After incubation at 37°C for 48 h, the bacterial load is expressed in CFU per unit mass of bone marrow. Samples of the bone marrow of the positive and negative control rabbits were also made.

The treatment of animals started 72 h after inoculation (day 3), and all five types of treatment were done twice a day, intramuscularly for 4 days. After 4 days of treatment (day 7), bone marrow samples were taken, and the bacterial count was evaluated.

On the 14^th^ day, the animals were euthanized by intravenous injection of a lethal dose of 100 mg thiopental under the marginal vein of the ear [33], the proximal half of the tibia was dissected into aseptic conditions, and bone marrow samples were taken. The bacterial load was then evaluated in the same way as on the third day and the seventh day.

Rectal temperature was taken on days 0, 3, 7, and 14 using a digital thermometer. The individual weighing was carried out on days 0, 3, 7, and 14 using a digital scale.

### 2.10. Statistical Analysis

The results were expressed as mean values ± SEM (standard error of the mean). A statistical analysis of the data was performed with a one-way analysis of variance followed by Tukeyʼs Multiple Comparison Test (ANOVA followed by Tukeyʼs test) (Graph Pad Prism, version 5.03). Differences of *p* < 0.05 were considered statistically significant.

## 3. Results

### 3.1. Minimal Inhibitory Concentrations

The AMC, gentamicin, and 1,8-cineole MIC values were shown in Table 2. The AMC has the lowest MIC for MSSA_2_. Gentamicin has the lowest MIC for MSSA_1_. And 1,8-cineole has the lowest MIC for MSSA_1_.

### 3.2. Effect of the Combination of Antibiotics and 1,8-Cineole

The effect of the two antibiotics tested against three strains of *S. aureus* by combining the two antibiotics, on the one hand, and combining each one of them with 1,8-cineole, on the other hand, was shown in Tables 3, 4, and 5. The combination of AMC and gentamicin gave no synergistic effect; however, an additive effect was noted for the MRSA and MSSA_2_ strains. For the combination of antibiotics with 1,8-cineole, a total synergistic effect is noted for the three strains combining AMC with 1,8-cineole, while the combination of gentamicin with 1,8-cineole showed a total synergistic effect for the MRSA strain and a partial synergistic effect for the other two MSSA strains.

### 3.3. Body Temperature

**Figure 1:**
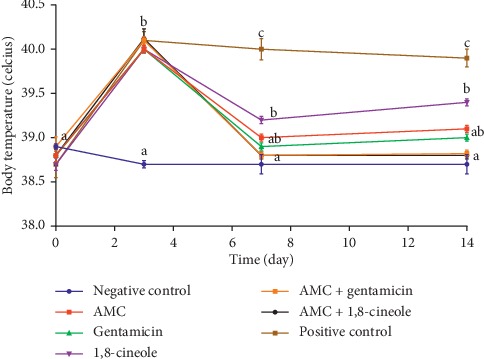
Changes in body temperature of rabbits. The values followed by different letters are significantly different from each other at *p* < 0.05.

Figure 1 shows the evolution of the rabbit's body temperature of different groups on days 0, 3, 7, and 14. It illustrates that, after inoculation, an increase in the body temperature of all groups of rabbits was observed except for the uninfected one. The temperature was around 38.6°C for all the rabbits at the beginning of the experiment, while it exceeded 40°C on day 2 for the groups of infected animals. During four days of treatment, the body temperature of treated rabbits decreased gradually reaching almost the normal temperature. In contrast, the body temperature of infected untreated rabbits remained above 40°C.

### 3.4. Body Weight

Table 6 shows the evolution of the rabbit's weight during the experiment. For the groups of infected animals, a weight loss during the three days (day 0–day 3) was noted. However, during the four days of treatment (day 3–day 7), an increase in weight of treated rabbits was observed regardless of the type of treatment, with no significant difference between the groups of treated animals. At the end of the experiment (day 14), the weights of animals from both the negative control and the AMC + 1,8-cineole group were significantly greater (*p* < 0.05) than those of the other groups.

### 3.5. Evolution of the Bacterial Load in the Bone Marrow

**Figure 2:**
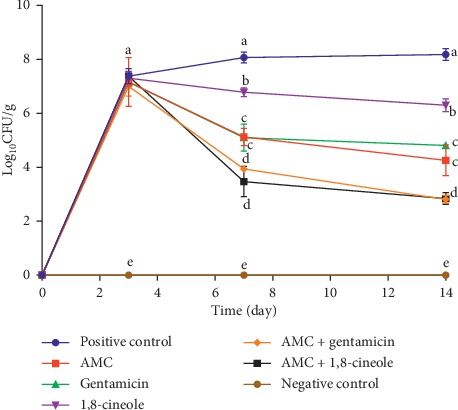
Evolution of the bacterial load in bone marrow in log_10_ CFU/g. The values followed by different letters are significantly different from each other at *p* < 0.05.

The evolution of the bacterial load in the bone marrow of all groups of animals is shown in Figure 2 and Table 7. Three days after inoculation, the bacterial load was around 10^7^ CFU/g for all groups except the uninfected group. During the four days of treatment (day3–day7), a decrease in the bacterial load was noted and was very significant for the groups of animals treated with AMC + 1,8-cineole, followed by the group treated with AMC + gentamicin, while a moderate decrease was observed for groups treated by AMC, gentamicin, or 1,8-cineole alone. A slight increase in bacterial load was noted for the group of infected untreated animals. During the second week of the experiment, and despite discontinuation of treatment, the bacterial load continued to decrease slightly in all five treated groups. *α*: the efficacy measurement was made by comparing the bacterial load before (day 3 after infection) 236 and after antibacterial therapy (day 7 after infection) (Table 7).

## 4. Discussion

Antibiotic treatment of osteomyelitis remains a clinical challenge [34]. This treatment is confronted with the increasing prevalence of multiresistant bacteria, particularly methicillin-resistant *S. aureus* MRSA [35]. Hence, there is an interest in finding alternatives to overcome the growing resistance of *S. aureus* strains to antibiotics.

The MIC values of AMC and gentamicin obtained are lower than those reported by Entenza et al. [36] with other strains of *S. aureus*. This difference is probably due to the use of different techniques; Entenza et al. used the macrodilution method with a higher inoculum of 10^7^ CFU/mL. Indeed, the bactericidal activity of antibiotics decreases when the inoculum increases, especially for *S. aureus*. The bacterial growth phase is also an important parameter that influences the antibacterial activity of antimicrobial agents [37].

The MIC of gentamicin obtained for the MRSA strain (2 *μ*g/mL) confirms the results of the susceptibility test by the disc diffusion method in which gentamicin resistance was found according to EUCAST [38]. For the AMC, the MICs determined by the microdilution were 1 *μ*g/mL, 0.5 *μ*g/mL, and 0.25 *μ*g/mL for MRSA, MSSA_1_, and MSSA_2_, respectively. Low MIC (≤1 *μ*g/mL) was obtained by Barry on 4.5% among 397 of cefoxitin-resistant staphylococci strains [39]. Methicillin resistance is mediated by an additional PBP (PBP2a) with low affinity for beta-lactam agents and it confers resistance to methicillin as well as to other beta-lactam antibiotics [40]. However, no clinical breakpoints were available for the AMC [41]. With regard to 1,8-cineole, the MIC values obtained are 16 mg/mL for MSSA_1_ and 32 mg/mL for MRSA and MSSA_2_. These values are lower than those obtained by Silva et al. [42] who obtained a MIC of 50 mg/mL for the *S. aureus* strain. Also, Mulyaningsih et al. [26] obtained a higher MIC value of 64 mg/mL, using the microdilution method with an inoculum of 5 × 10^5^ CFU/mL. This could be explained by the fact that the dispersion of EOs using either dimethyl sulfoxide (DMSO) or Tween 80 is known to reduce their antimicrobial activity. Indeed, our laboratory has already demonstrated that detergents such as Triton-X100 and Tween 80 or solvents such as ethanol decrease the antimicrobial effect of EOs or MICs [21]. The use of agar at 0.2% as a dispersing agent in this study explains the lower MICs obtained.

In order to measure the inhibitory activity of the interaction between AMC, gentamicin, and 1,8-cineole, the checkerboard assay by determining the fractional inhibitory concentration (FIC) was used. Langeveld et al. [19] reported that the checkerboard assay is the most frequently reported assay method for testing for synergy between antimicrobial substances. The combination of AMC and gentamicin showed no synergistic effect against the three strains tested, whereas amoxicillin/clavulanic acid and gentamicin are used in combination in the case of osteomyelitis caused by *Staphylococcus aureus* [43, 44]. In a recent study, Rondevaldova et al. [45] tested the effect of the combination of amoxicillin and demethyltexasin (DT) on 4 strains of *S. aureus*. A synergistic effect was obtained against three strains of MRSA, while no interaction was noted for the susceptible strain. Another study reported that the combination of gentamicin and daptomycin showed a synergistic effect on only 5% of isolates among eighty *S. aureus* tested [46].

Regarding the combination of each antibiotic with 1,8-cineole, a total synergistic effect was obtained when the AMC was combined with 1,8-cineole with a MIC four times lower. For gentamicin, its combination with 1,8-cineole induced a total synergistic effect for the MRSA strain with a 4-fold reduction of MIC, while a partial synergy was obtained for the other two strains. Many studies reported that the combination of EOs with antibiotics has a synergistic effect against microorganisms [20, 47–51]. Plant extracts in association with conventional antibiotics also reported a decrease of antibiotic MIC [49, 51]. This synergistic interaction appeared to be due to various mechanisms including sequential inhibition of common biochemical pathways and inhibition of protective enzymes [47]. Furthermore, the association of natural and synthetic drug induced a double attack on different target sites of bacteria which lead to an additive or synergistic effect [47].

The results obtained in the *in vitro* study encouraged us to perform the *in vivo* test. And so, we decided to test the effect of 1,8-cineole associated with AMC, compared with AMC associated with gentamicin and AMC alone, because the 1,8-cineole showed a total synergistic effect against *S. aureus* when it was associated with AMC. To our knowledge, this is the first time that 1,8-cineole has been used for this purpose.

The experimental model MRSA osteomyelitis in rabbits was used by Soranglou et al. [52] to evaluate the efficacy of intramuscular moxifloxacin, as well as by Taghipour et al. [7] in a comparative study of the effect of vancomycin, enrofloxacin, and trimethoprim/sulfamethoxazole. Bacterial inoculation is more often performed directly by intra-articular injection [33, 53]. We used the transcutaneous route to perform trepanning with a biopsy needle followed by injection of a suspension of *S. aureus* directly into the tibia. The inoculum size used was 10^9^ CFU/mL. It is the same as that used by Gaudin et al. [31] who developed an experimental model of acute osteomyelitis in rabbits and had a high bacterial load in the bone marrow, allowing the infection to persist for at least 14 days. After inoculation of the animals, the bacterial loads reached range from 10^7^ to 10^8^ CFU/g in bone marrow. This confirms that this experimental model is useful to evaluate the *in vivo* activity of antibacterial agents in bone infection.

For the treatment pathways of animals, we chose the intramuscular route rather than intravenous route. Our choice can be explained by the fact that intravenous drug administration in rabbits is extremely challenging due to the lack of available veins, and it is not possible to maintain an intravenous catheter for a long time in a vigilant rabbit. Moreover, intramuscular administration of antibiotics can result in peak serum concentrations within minutes at levels comparable to those observed after intravenous injections [52]. During the experiment, we monitored not only the bacterial load in the bone marrow but also body temperature and body weight. The results obtained showed that after inoculation, there is an increase in temperature and a loss of weight for the infected rabbits. Weight loss can be caused by a lack of appetite resulting from stress during the establishment of the model. However, the treatment of rabbits, especially with AMC associated with 1,8-cineole, is followed by a return of body temperature to its normal value and a weight similar to that of uninfected animals by the end of the 14th day. These results showed that these parameters could be useful for monitoring osteomyelitis. In the development of an MRSA animal model of osteomyelitis, Helbig et al. [54] monitored infection by measuring body temperature and body weight in combination with other parameters.

After four days of treatment, the bacterial loads in bone marrow showed that AMC in combination with either 1,8-cineole or gentamicin had the same efficacy with a percentage reduction of 99.99% and 99.98%, respectively, by the end of the experiment. This efficiency is significantly superior to that obtained with the AMC alone (99.81% of reduction) or gentamicin alone (99.49% of reduction). The treatment with 1,8-cineole alone showed the lowest percentage of reduction (89.91%).

Rondevaldova et al. [45] have reported that among the possible strategies for treating *S. aureus*-related diseases, the simultaneous administration of at least two antibiotics is often used. This simultaneous use of antibiotics is effective in extending their spectrum. However, it leads to the emergence of several multiresistant strains [55, 56], especially in hospitals where there is a great amount of pressure to select resistant strains by commonly used antibiotics [57]. Hence, there is an interest in substituting one of the two antibiotics by a natural antimicrobial agent such as 1,8-cineole. This substitution can lead to a reduction of the minimum effective dose of drugs, thus minimizing their possible toxic side effects and combating the resistance phenomenon with a lower treatment cost [58].

In this study, the enhancement of the activity of AMC was observed when it is associated with 1,8-cineole. Remmal and Akhmouch reported that cineole makes it possible to increase the efficacy of amoxicillin [28]. Specifically, they have demonstrated that the combination of amoxicillin and cineole makes it possible to obtain a synergistic effect which considerably reinforces the antibacterial activity of amoxicillin. Remmal and Akhmouch explained this by the fact that, in the presence of 1,8-cineole, stable amoxicillin complexes comprising at least three amoxicillin molecules form and protect the antibiotic against the action of *β*-lactamases in resistant bacteria. Additionally, 1,8-cineole has the capacity to destabilize the cell membrane or to affect cell respiration [49]. Therefore, its association with antibiotics can simultaneously act on different target sites, therefore, improving the observed results when compared to the antibiotic effect alone.

## 5. Conclusion

To our knowledge, this is the first in-depth study of the combination of AMC and gentamicin antibiotics with 1,8-cineole against *S. aureus*. Our results show that boosting the antimicrobial effect of antibiotics using 1,8-cineole appears to be a promising approach to investigate new pathways in the development of new antimicrobial drugs.

## Figures and Tables

**Table 1:** Resistance profile of the strains used.

Bacterial strains	Antibiotic resistance profile
MRSA	Penicillin, cefoxitin, gentamycin, erythromycin, norfloxacin, cotrimoxazole, trimethoprim-sulfamethoxazole, citric acid, tobramycin, and tetracycline
MSSA_1_	Penicillin and tetracycline
MSSA_2_	Susceptible

**Table 2:** MIC values.

*S. aureus* strains	MIC		
	AMC (*μ*g/mL)	Gentamicin (*μ*g/mL)	1,8-Cineole(mg/mL)
MRSA	1	2	32
MSSA_1_	0.5	0.5	16
MSSA_2_	0.25	1	32

**Table 3:** Effect of the combination of AMC with Gentamicin.

*S. aureus* strains	AMC (A)			Gentamicin (B)			FICI	Type of interaction
	MIC alone	MIC combined	FIC (A)	MIC alone	MIC combined	FIC (B)		
MRSA	1	0.5	0.5	2	1	0.5	1	Additive
MSSA_1_	0.5	0.5	1	0.5	0.25	0.5	1.5	No interaction
MSSA_2_	0.25	0.125	0.5	1	0.5	0.5	1	Additive

**Table 4:** Effect of the combination of AMC with 1,8-cineole.

*S. aureus* strains	AMC (A)			1,8-Cineole (B)			FICI	Type of interaction
	MIC alone	MIC combined	FIC (A)	MIC alone	MIC combined	FIC (B)		
MRSA	1	0.25	0.25	32	4	0.125	0.375	Synergy
MSSA_1_	0.5	0.125	0.25	16	4	0.25	0.5	Synergy
MSSA_2_	0.25	0.062	0.25	32	2	0.062	0.312	Synergy

**Table 5:** Effect of the combination of gentamicin with 1,8-cineole.

*S. aureus* strains	Gentamicin (A)			1,8-cineole (B)			FICI	Type of interaction
	MIC alone	MIC combined	FIC (A)	MIC alone	MIC combined	FIC (B)		
MRSA	2	0.5	0.25	32	8	0.25	0.5	Synergy
MSSA_1_	0.5	0.25	0.5	16	4	0.25	0.75	Partial synergy
MSSA_2_	1	0.5	0.5	32	8	0.25	0.75	Partial synergy

**Table 6:** Evolution of body weight. The values followed by different letters are significantly different from the values of the positive control group at *p* < 0.05.

Groups							
Time (day)	Positive control	AMC	Gentamicin	1,8-cineole	AMC + gentamicin	AMC + 1,8-cineole	Negative control
Day 0	1473 ± 157a	1450.33 ± 11a	1450.33 ± 118.44a	1443.66 ± 109.56a	1531.33 ± 179a	1735.33 ± 149a	1513 ± 128a
Day 3	1419 ± 16a	1401.67 ± 113a	1401.66 ± 119.56a	1395 ± 115a	1476 ± 189a	1687 ± 142a	1596.33 ± 132a
Day 7	1458.67 ± 114a	1497.33 ± 113a	1497.33 ± 119.78a	1487.33 ± 113a	1594 ± 182a	1807 ± 148a	1729.67 ± 140a
Day 14	1568.33 ± 135a	1660.67 ± 140a	1660.66 ± 147.56a	1650.66 ± 140a	1777 ± 147a	2002 ± 145b	1973 ± 148b

**Table 7:** Difference in bacterial load in the bone marrow between day 3 and day 7. The values followed by different letters are significantly different from each other at *p* < 0.05.

Groups	Mean ± SD log10 CFU/g (day 3–day 7)^*α*^
Positive control (*n* = 6)	(0,68 ± 0,48) b
AMC (*n* = 6)	(−2,04 ± 0,47) d
Gentamicin (*n* = 6)	(−2,06 ± 0.49) d
1,8-cineole (*n* = 6)	(−0.56 ± 0.06) c
AMC + Gentamicin (*n* = 6)	(−3,40 ± 0,4) e
AMC+1,8-cineole (*n* = 6)	(−3,43 ± 0,41) e
Negative control (*n* = 6)	(0 ± 0) a

## Data Availability

The data used to support the findings of this study are available from the corresponding author upon request.
